# 2-Hydroxypropyl-β-Cyclodextrin Aggregates: Identification and Development of Analytical Techniques

**DOI:** 10.3390/ma11101971

**Published:** 2018-10-13

**Authors:** André Rodrigues Sá Couto, Alexey Ryzhakov, Thorsteinn Loftsson

**Affiliations:** Faculty of Pharmaceutical Sciences, University of Iceland, Hofsvallagata 53, IS-107 Reykjavik, Iceland; ars70@hi.is (A.R.S.C.); alexey@hi.is (A.R.)

**Keywords:** 2-hydroxypropyl-β-cyclodextrin, aggregation, critical aggregation concentration, permeation, nanoparticles

## Abstract

It is extremely important for pharmaceutical formulators to have analytical methodology that provides efficient detection and quantification of HPβCD aggregates. Five different methods were then evaluated for their potential to detect these aggregates and to determine critical aggregation concentration (cac): osmometry, viscometry, tensiometry, dynamic light scattering (DLS), and permeability studies. Overall, tensiometry was an inadequate method with which to study HPβCD aggregation, since the addition of HPβCD to water resulted in only minor changes in surface tension. Osmolality and viscosity studies have shown that for HPβCD, solute–solvent interactions are the main contributors for the observed deviation from ideality. These deviations might be related to the presence of aggregates. The DLS method proved to be an effective method with which to detect HPβCD aggregates and estimate their hydrodynamic diameter, although it presented some limitations concerning their quantification. In terms of the assessed methods, permeation studies were shown to be the best to study HPβCD aggregation phenomena, since they were the only method where the detection of aggregates and the determination of apparent cac values was possible. Also, it was the least invasive for the HPβCD samples and the method that provided more conclusive data. Results suggested that HPβCD, as expected, has less tendency to form aggregates than βCD.

## 1. Introduction

It has been well documented that cyclodextrins (CDs), especially the natural ones, can self-assemble in aqueous solutions to form CD aggregates that are kept together by weak intermolecular forces [1,2,3,4].

Although the natural β-cyclodextrin (βCD) is the most available and most affordable CD [5], it has very low solubility in water (i.e., only 18.5 mg/mL at 25 °C) [6] and a high tendency to self-aggregate. Formation of βCD aggregates and various methods for their detection is described in several publications. For instance, Bonini et al. used dynamic/static light scattering and cryo-TEM techniques to detect βCD aggregates in aqueous solutions at βCD concentrations as low as 2–3 mM [2]. De Sousa et al., using particle size measuring techniques, reported similar but somewhat lower critical aggregation concentration (cac) values for βCD or approximately 1–2 mM [7]. Mixcoha and co-workers used computational molecular dynamic simulations to show the presence of βCD aggregates in aqueous solutions [8], and Coleman et al. showed that the aggregate size decreased and solubility of βCD increased with increasing pH of the aqueous media or when chaotropic solutes were added to the media [9]. Due to the low solubility and aggregation propensity of βCD and the other natural CDs (i.e., αCD and γCD), several water soluble derivatives of these CDs were synthetized [10]. Random substitution of the hydroxy-moieties of the natural CDs decreased their tendency to crystallize and disrupted the formation of intramolecular hydrogen bonds. The substitution with consequent changes in the physiochemical properties led to considerable solubility improvements [11]. One of the most relevant derivatives for the pharmaceutical field is 2-hydroxypropyl-β-cyclodextrin (HPβCD) [12]. Despite the fact that in theory HPβCD should be less prone to aggregate that βCD, some unexpected phenomena were observed with this βCD derivative when included in aqueous pharmaceutical formulations. Haüsler et al. described the presence of aggregates in aqueous HPβCD solutions and observed changes in CD aggregation profile when urea was added to the media [13]. Messner et al. verified by permeation studies and HPLC drug quantification formation HPβCD aggregates that consisted of drug/HPβCD inclusion complexes [4,14]. However, due to the physiochemical properties of CDs (they do not possess chromophore), they were unable to detect aggregates in pure aqueous HPβCD solutions. In order to apply the permeation method to detect HPβCD aggregates, a new type of HPLC detector had to be used. Here, a charged aerosol HPLC detector is used to detect HPβCD in aqueous media.

The objective of the present work is to develop a suitable/reliable analytical method to study the aggregation phenomena in pure aqueous HPβCD solutions and to determinate the cac value.

## 2. Results and Discussion

### 2.1. Validation of UHPLC Method

Due to the lack of chromophores or fluorophores groups, detection of CDs by HPLC using conventional detectors (UV/Visible detectors or fluorometric detectors) was challenging. Recent advances in HPLC detector technologies have overcome this limitation and, nowadays, detectors such as charged aerosol detector (CAD), evaporative light scattering detector (ELSD), and refractive index (RI), among others, are used to detect CDs in different kind of media [15]. A CAD detector was selected for the HPβCD detection, as it is more sensitive than RI and ELSDs detectors, even though CAD has a similar detection mechanism to ELSD.

The linearity between the peak area and the individual HPβCD concentrations on log-log scale was assessed. The tested method proved to be linear for HPβCD over the studied concentration range (0.0008–1% *w*/*v*). The obtained results also showed a good correlation factor of 0.9993. Using Equations (5) and (6) LOD and LOQ were calculated and found to be 0.00014% and 0.00042% (*w*/*v*), respectively. To determine these two values, three different concentrations of HPβCD were prepared, namely, 0.5%, 2.5%, and 5% *w*/*v*. All assessed CD concentration had to be diluted with water prior to injection. The percentages of recovery (accuracy) and intraday precision (repeatability) of the selected CD concentrations are shown in Table 1.

### 2.2. Osmolality Measurements and Activity Coefficient Determination

Intermolecular interactions caused by London-van der Waals forces and H-bonding result in significant deviations in system behavior from ideality. In order to evaluate such deviations, the method of activity coefficient can be applied. Numerous publications devoted to the non-ideal behavior of saccharides solutions were based on this method. The analysis of colligative properties of galactose [16], glucose [16,17,18], maltose [17], maltotriose [17], mannose [16], and sucrose [18,19,20] have shown the same trend: activity coefficient increased with increasing saccharides concentration, corresponding to a positive deviation from ideality. In majority of the cases, authors explained such behavior as strong interactions between sugar and water molecules.

**Figure 1:**
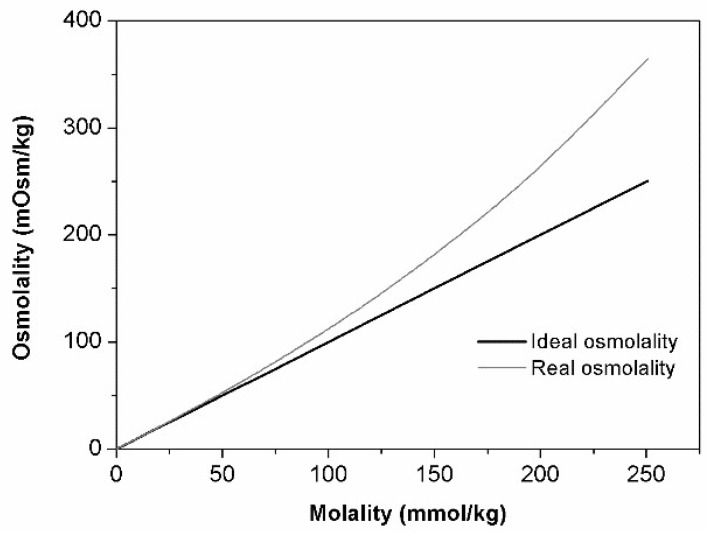
Concentration dependencies of osmolality for HPβCD.

Recently [21], we have analyzed osmometry results of native CDs (αCD and βCD) and obtained data pointed to their propensity to self-association (negative deviation from ideality), which is not common for saccharides. In current studies, we would like to know which type of non-ideality behavior HPβCD follows. For that purpose, calculation of activity coefficient was carried out. The concentration dependency of osmolality for HPβCD solutions is presented in Figure 1.

Activity coefficients were derived from the following Equation:

(1) $${\ln\gamma }_{2}\;=\;\left(\phi \;-\;1\right)\;+\;\overset{m}{\underset{0}{\int }}\frac{\phi \;-\;1}{m}\mathrm{dm}$$

in which γ_2_ is HPβCD activity coefficient; m is molality of HPβCD solution; and ϕ is osmotic coefficient, which was obtained as proportional coefficient between osmolality ($\bar{m}$) and molality (assuming that HPβCD does not dissociate in water, n = 1):

(2) $$\bar{m}=n\phi m$$

In order to solve integral part in Equation (1), osmotic coefficient was presented as polynomial function of the type

(3) $$\phi \;=\;1\;+\;\overset{n}{\underset{i\;=\;1}{\sum }}A_{i}m^{i}$$

**Figure 2:**
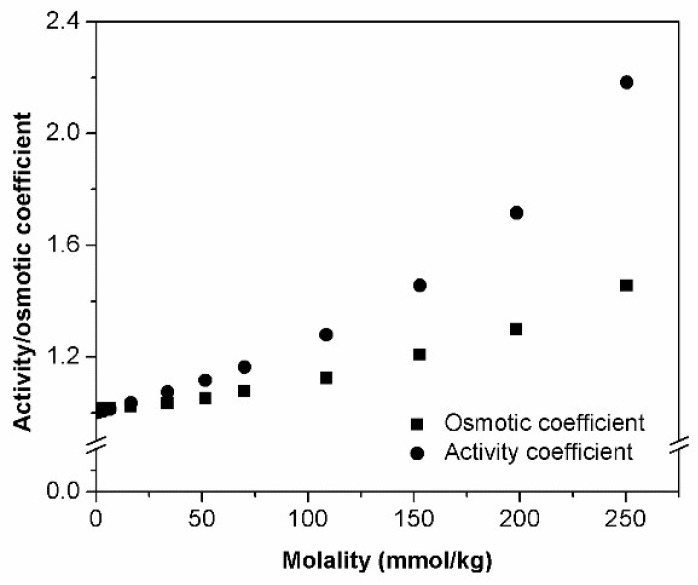
Variation of osmotic/activity coefficients with HPβCD concentration.

Concentration dependencies of osmotic and activity coefficients are illustrated in Figure 2. Calculated activity coefficients obey following empirical Equation:

(4) $$\gamma_{2}\;=\;{56.81\;m}^{3}\;-\;{5.00\;m}^{2}\;+\;2.40\;m\;+\;1$$

As it can be seen on Figure 2, HPβCD aqueous solutions represent the example of positive deviation from ideality—the higher the concentration of solute, the higher the osmotic/activity coefficient. Such behavior can be attributed to two factors that may be concurrent or not. On the one hand, the presence of HPβCD isomers might overly enhance the molecular randomness of solution opposing the tendency to freezing. Therefore, addition of HPβCD causes significant lowering of the freezing point and, consequently, positive deviation of osmolality. Such assumption can be supported by the conclusion that was made by Miyajima and co-workers [16] based on analysis of partial molar entropy of water at increase monosaccharides’ concentration. On the other hand, the decrease of water molecules’ mobility can be caused by strong solute–solvent interactions that might take place in HPβCD solutions. Similar observations/conclusions are also characteristic for saccharides [17,18,20]. The same presented arguments can possibly explain the high viscosity values registered for HPβCD solutions.

Contrary to what was reported for native CDs and following the same tendency of saccharides, the results of HPβCD pointed to the existence of stronger solute–solvent interactions when compared to solute–solute interactions.

Similar conclusions can be found in previous publications where authors also described a positive deviation from ideality for HPβCD [12,22], as well as other CD derivatives: DMβCD [23], SBEβCD [12,22], HPγCD [12,22], and SBEγCD [12].

Overall, obtained results cannot completely exclude the presence of self-aggregated particles (solute–solute interactions); however, in this case, solute–solvent interactions were the main contributors to the observed non-ideal behavior.

### 2.3. Viscosity Measurements

**Figure 3:**
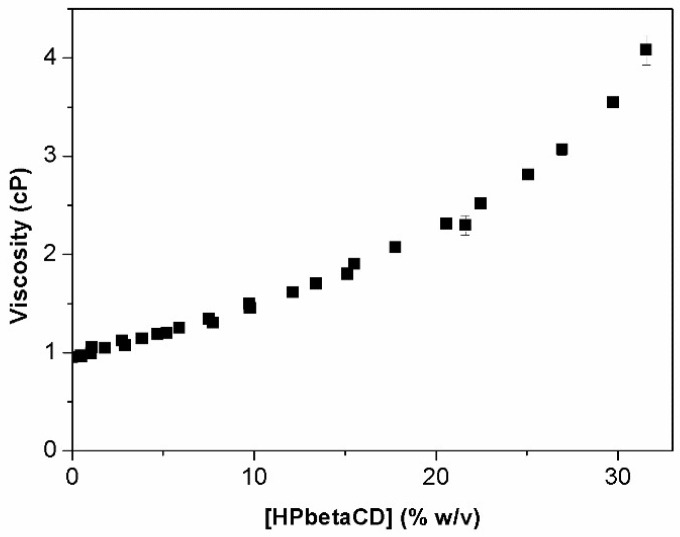
Increase in viscosity of pure aqueous HPβCD solutions with increasing HPβCD concentrations at 25 °C.

Figure 3 illustrates of HPβCD viscosity behavior with increasing concentration. A linear increase of viscosity is observed at HPβCD concentration below 11% (*w*/*v*), after which a positive deviation from linearity is observed. This upward curvature at higher HPβCD concentrations is not a novel observation, and similar results have also been described before [13,24,25,26,27].

Viscosity is a measure of the free movement of water molecules. The linear relationship described above is explained by a linear increase of water networking with increase of HPβCD concentration. The decrease in water molecules mobility due to their binding by HPβCD leads to an increase of media viscosity [28]. The exponential rapid increase of viscosity above 11% (*w*/*v*) HPβCD is probably due to the ability of HPβCD to behave as a structure maker [25] and the consequent HPβCD self-aggregation and overlapping of hydration shells [25,28]. Also, the entrapped water molecules inside of the formed aggregates can increase their effective hydrodynamic volume, which will also contribute to the increase of apparent viscosity [29]. The inflexion point was calculated from Figure 3 and determined to be approximately 11% (*w*/*v*) HPβCD. This value is quite similar to the cac value for aqueous HPβCD solutions (i.e., 11.8%) obtained from permeation studies with the smallest pore size used (MWCO 3.5–5 kDa). The similarity of the two values indicates that the exponential increase in viscosity with increase of HPβCD concentration can be related to the self-assembly process.

Haüsler et al. made similar observations and reported that HPβCD molecules had tendency to associate. Furthermore, they added different amounts of urea, which is a chaotropic agent, and observed a viscosity decrease due to disruption of aggregates and consequent increase in water mobility [13]. Although the attempt to determine cac values applying this method was not successful, this parameter was applied during particle size measurements in aqueous CD solutions.

### 2.4. Surface Tension Measurements

**Figure 4:**
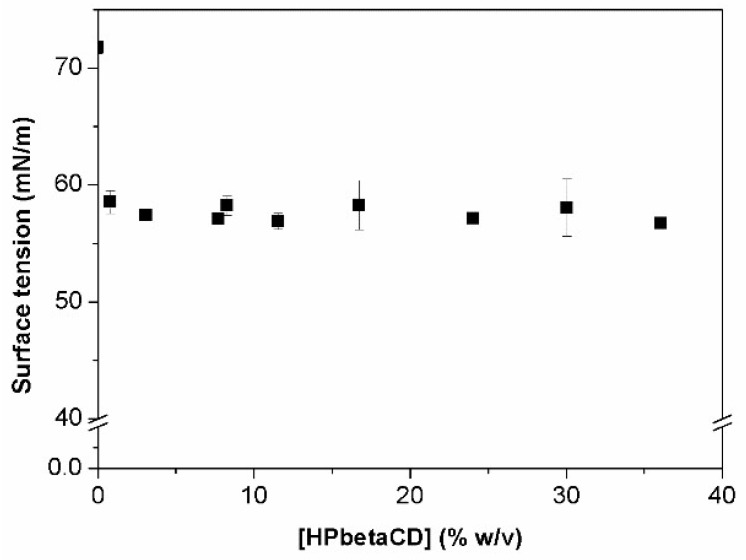
Surface tension variation with concentration of HPβCD aqueous solutions at 25 °C.

Figure 4 shows that the addition of HPβCD to water results in a small decrease in the surface tension. This small decrease in surface tension is in agreement with earlier observations by various investigators [24,26,30,31].

A decrease of 14 mN/m was observed at 0.5% (*w*/*v*) HPβCD concentration (corresponding to CMC), after which constant values are obtained even at HPβCD concentration as high as 35% (*w*/*v*). Yoshida et al. suggested that this decrease in surface tension is due to the surface activity of HPβCD [24,30]. Other substituted βCDs also possess surface activity greater than that of the natural βCD [13,26,31]. The stronger interaction of hydroxypropyl groups with water molecules is probably responsible for the disruption of the water network at the surface. HPβCD results in positive deviation from linearity of the activity coefficient, indicating that solute–solvent interactions are preferred over solute–solute interactions. This can explain why HPβCD promotes a slight change in surface tension. However, here the focus is on the detection of CD aggregates and how these structures could influence or modify the surface tension of a solution when HPβCD is present.

The results show that tensiometry does not appear to be a usable method for the detection of aggregates. The cac value cannot be related to the CMC value, as this is really low (~0.5%), and this concentration as aggregation starting point for a substituted CD does not seem to be realistic.

### 2.5. Size Distribution of HPβCD Particles

**Figure 5:**
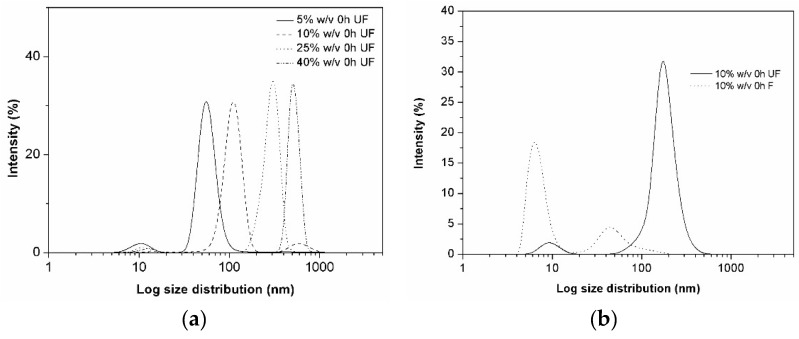

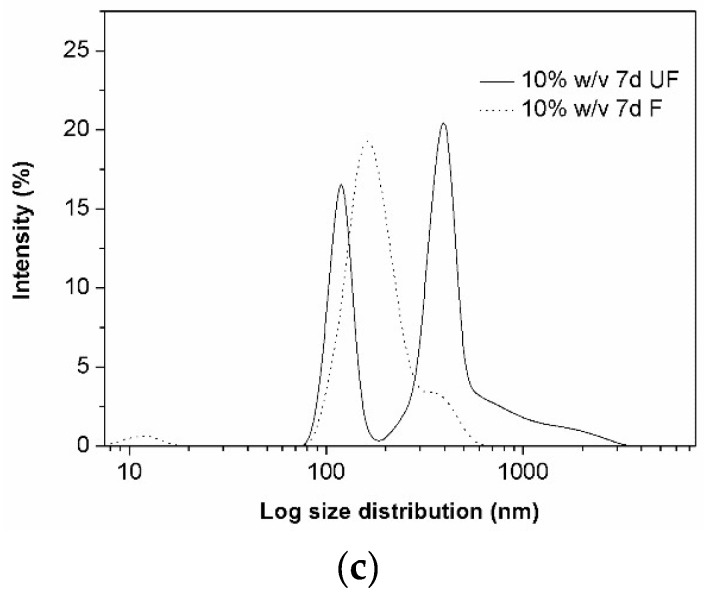
(**a**) Intensity fraction size distributions of the apparent hydrodynamic radius of HPβCD aggregates in pure aqueous HPβCD solutions, as measured by DLS at 23 °C. (**b**) Particle size distribution of HPβCD aggregates in freshly prepared aqueous solutions and after equilibrium of the sample solutions for seven days at room temperature (about 23 °C) (**c**).

In pure aqueous HPβCD solutions, the scattering intensity at concentrations below 1% (*w*/*v*) was very low, meaning that, if present, particles cannot be detected accurately. At higher HPβCD concentrations, particles and, especially, aggregates could readily be observed. Figure 5a shows aggregate size distribution in freshly prepared solutions (measured right after their preparation without filtration). This figure clearly shows that particle size increased with increasing HPβCD concentration. In all tested HPβCD solutions, bimodal populations were observed. The smaller populations with hydrodynamic size of about 10 nm might present HPβCD monomers or very small aggregates. The relative contribution of this population size decreases with increasing HPβCD concentration. The hydrodynamic diameter of the larger aggregate populations and their intensity increase with concentrations from about 80 nm at 5% (*w*/*v*) HPβCD to about 800 nm at 40% (*w*/*v*) HPβCD. Initially, small aggregates are being formed, and then as the HPβCD concentration increases, both their size and abundance increases.

The effect of filtration and storage time on the aggregate size distribution was also studied (Figure 5b,c). For this purpose, the 10% (*w*/*v*) HPβCD solution in water was filtrated through 0.45 µm membrane filter and then measured immediately after preparation (at time zero) and again after seven days’ equilibration without agitation at room temperature. The reference solution was the unfiltered 10% (*w*/*v*) HPβCD solution.

Figure 5b shows that immediately after filtration (i.e., at time zero), the particle size was considerably smaller in the filtered (F) than in the unfiltered (UF) solution. Both the F and UF solutions had similar bimodal aggregate size population but were comprised of different sizes. Just after preparation of samples and before filtration, a small population of particles (monomers, dimers) around 10 nm is observed, along with a second population of larger aggregates with hydrodynamic diameter of about 200–300 nm. After filtration through 0.45 µm pore size membrane filter, the same bimodal profile is registered but with a different size distribution. The sample filtration disassembled the larger aggregates with a consequent formation of smaller aggregates with hydrodynamic diameter of about 80 nm. Furthermore, the filtration resulted in a large increase of monomer population, and this population has higher intensity than the aggregate population.

Figure 5c presents the size distribution after equilibrium of the sample solutions for seven days. A shift to the right is observed showing formation of larger aggregates due to further aggregation in the unstirred HPβCD solution with time. The size of particles increased significantly for the UF samples that, however, maintained their bimodal size distribution with size populations of about 100 nm and 800 nm. The size distribution of the F solutions also changed, showing one major size population of about 200 nm. These results show that filtration reduced the hydrodynamic diameter of the HPβCD aggregates, and that storage increased the aggregate diameter. Previously, other investigators have reported similar observations for the natural CDs [2,3]. Furthermore, the study shows that the HPβCD aggregation is a reversible process.

The light scattering analytical method can be a reliable method to describe and study self-aggregation of CDs, but the results depend on several variables. For example, the angle used for determining the hydrodynamic diameter will affect the results [32]. Also, guest compounds will affect the aggregation and the size of the aggregates, and they will depend on if a single CD system is being studied or if an effect of some other molecules on the CD aggregation is being investigated. Consequently, the previously reported cac values for pure HPβCD [32] and HPβCD complexes [33,34,35] can be different from the values obtained in this study. In this present study, the DLS equipment used did not allow determination of the cac value.

### 2.6. Permeation Studies

#### 2.6.1. Optimization of Permeation Studies

Before determination of the HPβCD aggregation profile by Franz diffusion cells experiments, it is important and crucial to know and optimize some of the experimental parameters, namely, determination of the time that is needed to obtain steady state HPβCD permeation through the cellulose semi-permeable membrane (i.e., the lag-time) and duration of the steady state permeation.

HPβCD steady state permeation was obtained at 0.20 h (12 min). After 0.25 h (15 min), a straight line is obtained with a slope representing the flux (*J*) in Equation (7). With this information, the permeation studies for HPβCD could be planned, starting the first collection point at 15 min and the last one at 75 min.

#### 2.6.2. Determination of Flux and Apparent cac Values for HPβCD

For each HPβCD concentration samples were collected every 15 min after the lag time (15 min) had been reached with time 0 when the donor phase (i.e., the HPβCD solution) was applied to the membrane surface (i.e., to the donor chamber). Samples were analyzed by UHPLC, and graphs describing mass of CD permeated (mg) versus time (s) were drawn. From these graphs, slopes (dq/dt) for each sample concentration were registered and used to determine CD flux (J) using Equation (7) described on materials and methods section.

**Figure 6:**
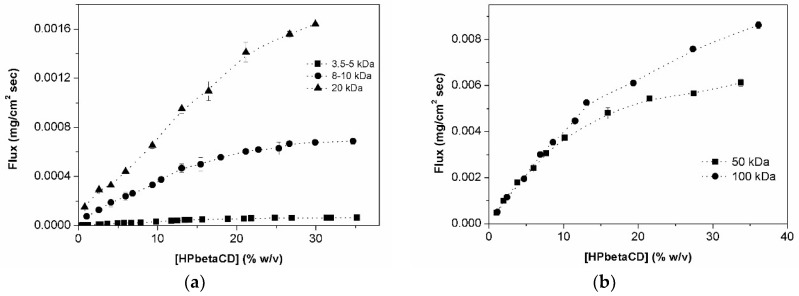
Flux profile of HPβCD through semipermeable membranes: 3.5–5, 8–10, and 20 kDa (**a**) and 50, 100 kDa (**b**) at room temperature (about 23 °C).

Figure 6a shows the flux of HPβCD versus its concentration through three semi-permeable membranes of different MWCO (i.e., 3.5–5, 8–10, and 20 kDa). Following Fick’s first law (see Equation (3)), one should expect a linear increase of flux with increasing HPβCD concentration, but this is not always the case. Initially, the graphs in Figure 6a display a linear section corresponding to free permeation of HPβCD monomers and possibly small aggregates. When concentration increases, both the amount and size of aggregates increase, affecting the ability of HPβCD to permeate the semi-permeable membrane. Furthermore, small aggregates (e.g., monomers, dimers, and trimers) will permeate the larger MWCO membranes. For this reason, an increase in apparent cac value is observed with increasing of MWCO or 11.8%, 14.3%, and 19.1% (*w*/*v*) for the 3.5–5, 8–10, and 20 kDa membranes, respectively. Also smaller particles are able to pass membranes with the larger MWCO, but aggregates larger than the pore size are the ones responsible for the observed negative deviation from Fick’s First law. This negative deviation has been observed also by different authors [1,15,27,36,37,38,39] which, like in our case, suggested formation of CD self-aggregates.

There are two simultaneous processes ongoing during the permeation studies through semi-permeable membranes: (1) a solute transfer from donor to receptor phase (simple diffusion) and (2) a solvent transfer in the opposite direction (osmosis). These two processes are not controlled by the same physicochemical properties. The osmotic effect depends on the MWCO of membrane (i.e., larger pore size results in faster diffusion for water molecules) and osmolality difference of the two phases; the larger this difference is, the larger volume of water that will be transferred from the receptor to the donor chamber. The HPβCD diffusion from the donor to the receptor chamber is influenced by the MWCO of membrane but also by other parameters such as aggregate size, concentration, and viscosity of the tested sample (i.e., the donor phase). All these properties can affect the observed HPβCD flux by diluting the HPβCD donor phase concentration or by perturbing the normal HPβCD permeation. For example, extremely high viscosity can prevent or delay the release of permeating molecules from one compartment to the other, and high substrate concentrations can increase the difference in osmolality between the two chambers and by this way increase osmotic pressure that can work as a counter flow force delaying permeation of molecules through the membrane.

**Figure 7:**
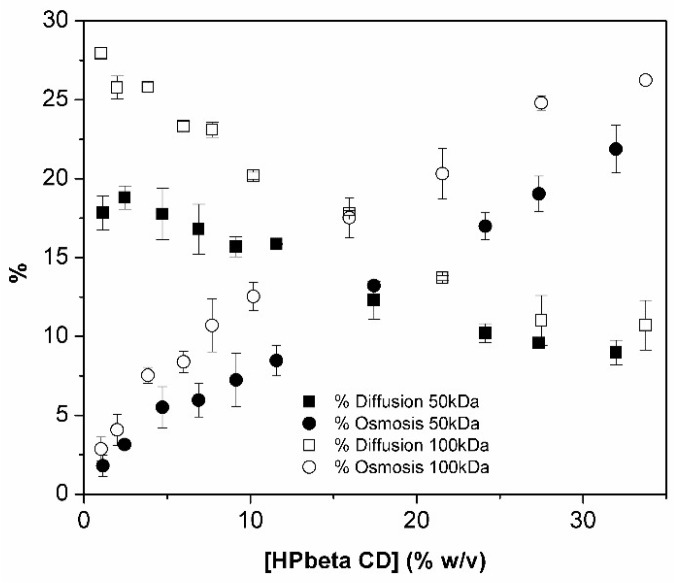
% osmosis (circles) and % diffusion (squares) determined for aqueous HPβCD solutions when studies used different MWCO semipermeable membranes: 50 kDa (black) and 100 kDa (white) (n = 3).

The analysis of HPβCD graphic for 50 and 100 kDa membrane (Figure 6b) shows similar profile behavior as displayed in Figure 6a. However, a more careful interpretation and analysis has to be performed. Despite the slight negative deviation obtained in the case of the 20 kDa membrane (Figure 6a), the profile is almost linear (indicates that diameter of the HPβCD aggregates is close to globular molecule of molecular weight of 20 kDa). Looking to the accentuated inflexion of both 50 and 100 kDa MWCO membrane curve, one might suppose that it is also due to aggregates size. However, the difference in osmolality between HPβCD (donor phase) and water (receptor phase) allied to the larger pore size of membranes makes one speculate that under such conditions probably the osmosis process is so fast that it acts like a counter flow force that prevents normal particle permeation with consequent donor phase dilution during the experiment. Also, viscosity of samples can contribute to this phenomenon. The donor phase dilution might be responsible for the negative deviation from linearity. To get better understanding of these phenomena, contribution of osmosis and diffusion for permeation process through the 50 and 100 kDa membranes were calculated. As observed in Figure 7, the point where osmosis starts to be higher than diffusion is coincident with the inflexion point on the flux curves of these membranes. This phenomenon does not affect the three other membranes with much lower MWCO.

These observations make us believe that under such conditions, we were not longer working under sink conditions [40], and so these two larger pore size membranes cannot be used to study HPβCD aggregation and determine apparent cac values.

From the intersection of the linear portions of the plots at concentrations above and below the inflection points, it was possible to determine the apparent cac (concentration after which one start to detect formation of aggregates) for HPβCD. The estimated apparent cac values for HPβCD in pure water are presented in Table 2.

Permeation showed advantages over the other methods tested with less variability and more precision and seems to be the most valuable method to determine HPβCD aggregates in water.

## 3. Conclusions

Several available analytical approaches were explored in an effort to develop a method that could be used to efficiently detect and quantify HPβCD self-aggregates in aqueous solutions. However, not all of them proved to be useful. Results from tensiometry studies showed that HPβCD had only a minor effect on the surface tension, and the conclusion was that this is not a good method with which to study the aggregation phenomena of HPβCD.

A positive deviation after the linear increase of media viscosity with HPβCD concentration was registered (at approximately 11% *w*/*v* HPβCD). This exacerbated increase is due to the binding of water molecules by HPβCD with consequent reduced mobility of water molecules that might also be related to formation of HPβCD aggregates. However, there are no scientific bases that enable correlation of this value with an apparent cac value.

Similarly to what has been described for saccharides, osmometry results show a positive deviation from ideality when plotting osmolality against HPβCD molality. An increase of osmotic and activity coefficient with increasing HPβCD concentration was also noticed, suggesting the existence of strong interaction between CD and water molecules. Although these interactions (solvent–solute) are probably favored over solute–solute interactions and are the main contributors to the observed osmolality behaviour, we cannot exclude the presence of HPβCD aggregates. This method did not prove to be suitable for aggregates’ quantification.

Contrary to previously published work [32], we could not determine the apparent cac value for HPβCD by a DLS method. Self-assembly process is a fast and dynamic equilibrium (formation/dissemble) and probably too fast to be detectable by the DLS machine used in this study. However, other types of important findings were made applying this method, such as the size range of HPβCD aggregates, the tendency for aggregates’ size to increase with increasing HPβCD concentration, and the important effect of filtration (affected the size distribution by disassemble of larger aggregates form smaller ones) and equilibration time (increasing size with time after filtration).

Permeability showed that aggregation and aggregate size increases with increasing HPβCD concentration with determined apparent cac value of 11.8% (*w*/*v*) for the 3.5–5 kDa MWCO membrane. The analysis of permeation results for HPβCD clearly indicates that the aggregates have hydrodynamic diameter larger than 20 kDa, as they do not permeate freely through the 20 kDa MWCO membrane. For the 50 and 100 kDa MWCO membranes, the flux still deviates from linearity, but one can speculate that is not only due to the aggregate particle size but also due to other factors such as high osmotic pressure and viscosity. HPβCD is less prone to self-aggregate than its precursor βCD, since the presented apparent cac values for HPβCD (starting from 11.8% *w*/*v*) were significantly higher than for βCD (starting from 0.8% *w*/*v*).

As CD aggregates are quite unstable and can be disrupted by forces applied in specific analytical techniques, to have an unstirred and quite still donor phase during permeation experiment is really important. Overall, the permeation method was the best method to evaluate HPβCD aggregation, as it provided more conclusive results and is the least “invasive” for the self-assembled samples.

The main results, advantages, and disadvantages of each analyzed technique are summarized in Table 3.

## 4. Materials and Methods

### 4.1. Materials

2-Hydroxypropyl-β-cyclodextrin (HPβCD) DS 0.62 (MW 1380 Da) was kindly provided by Janssen Pharmaceutica (Beerse, Belgium). Milli-Q water (Millipore, Billerica, MA, USA) was used to prepare CD solutions and mobile phases. Acetonitrile of HPLC grade was purchased from Sigma–Aldrich (St. Louis, MO, USA). A set of 5 different pore size Spectra/Por^®^ semipermeable cellophane membranes (Spectrum Europe, Breda, The Netherlands) with molecular weight cutoff (MWCO) of 3.5–5, 8–10, 20, 50, and 100 kDa were used for permeation studies.

### 4.2. Quantitative Determination of HPβCD

Quantitative determination of HPβCD was performed on a reverse-phase, ultra-high-performance liquid chromatography (UHPLC) Ultimate 3000 series system from Dionex Softron GmbH (Germering, Germany) consisting of a LPG-3400SD pump with a built-in degasser, a WPS-3000 autosampler (Dionex, Germering, Germany), a TCC-3100 column compartment (Dionex, Germering, Germany), and a CoronaR ultra RS detector (Dionex, Germering, Germany). During the stationary phase, a Phenomenex Kinetex C18 150 × 4.60 mm 5 µm column with a matching HPLC Security Guard (Phenomenex, Cheshire, UK) were used. The mobile phase consisted of equal volumes of acetonitrile and water; the flow rate was 1.0 mL/min, and the injection volume was 5 µL. Retention time and peak area of all chromatograms were determined using the software ChromeleonR version 7.2 SR4 (ThermoScientific, Waltham, MA, USA).

### 4.3. Validation of the UHPLC-CAD Method

The validation of the UHPLC analytical method for detection of HPβCD in aqueous solutions was performed according to the Q2(R1) guidelines from the International Conference on Harmonization Guidelines [41]. The parameters that were validated are as follows: linearity and range, repeatability, detection limit (LOD), quantification limit (LOQ), and accuracy. Aqueous HPβCD solutions of various concentrations were prepared through sonication at 60 °C for 60 min and then allowed to cool to room temperature. The water content of HPβCD powder was determined using an A&D MX-50 moisture analyser (A&D company, Limited, Tokyo, Japan) and accounted for.

#### 4.3.1. Linearity and Range

Aqueous solutions containing 0.001 to 1% (*w*/*v*) HPβCD were prepared in order to evaluate the linearity and range. Calibration curves were prepared in triplicate and each sample injected three times. Linearity of the curves obtained was analyzed by plotting logarithm of the peak area vs. logarithm of the CD concentration.

#### 4.3.2. Repeatability

Repeatability is the parameter that evaluates the variability of the assay being expressed in relative standard deviation (%RSD). To validate this parameter, three concentrations of HPβCD were randomly selected and injected six times. Acceptance criteria for this test were established as RSD ≤ 1%.

#### 4.3.3. Accuracy (% Recovery)

Accuracy was measured by injecting, three times, three different concentrations of the studied CD solution. Mean recovery (difference between theoretical and real concentration) was then calculated. In order to be accepted and validated, this parameter needed to be 100 ± 2% at each concentration [15].

#### 4.3.4. Limit of Detection (LOD) and Limit of Quantification Limit (LOQ)

These were obtained from the linearity test as follows [41]:

(5) $$\mathrm{LOQ}\;=\;\frac{\mathrm{Standard}\;\mathrm{Deviation}}{\mathrm{Slope}}\;\times \;10$$

(6) $$\mathrm{LOD}\;=\;\frac{\mathrm{Standard}\;\mathrm{Deviation}}{\mathrm{Slope}}\;\times \;3.3$$

### 4.4. Determination of the Water Content

Water of the HPβCD samples (approx. 5 g) was determined using an A&D MX-50 moisture analyzer (A&D company, Limited, Tokyo, Japan). The samples were evenly spread over the aluminium pans, in order to avoid uneven drying of the samples. The moisture content of HPβCD was measured in triplicate and results presented in average (%) ± standard deviation (SD). The water content of HPβCD was determined to be 6.21 ± 0.12%.

### 4.5. Osmolality Measurements (Cryoscopic Osmometry)

The osmolality studies were performed using Osmomat 030 (Gonotec GmbH, Berlin, Germany). Each point was measured at least three times, and the mean values and SD were calculated. MilliQ water was used to determine background, and standard NaCl solutions of 300 and 400 mOsm (Knauer GmbH, Berlin, Germany) were used for initially osmometer calibration. Recalibrations were performed between measurements.

### 4.6. Viscosity Measurements

The viscosity measurements of aqueous HPβCD solutions were performed with a Brookfield model DV-I + (USA) viscometer equipped with a Polystat circulating thermostatic water bath (Cole Palmer, Vernon Hills, IL, USA) set to 25 °C. All samples were analyzed in triplicate in order to calculate average values and SD.

### 4.7. Surface Tension Measurements

HPβCD solutions were measured using a Krüss K100 tensiometer (Krüss GmbH, Hamburg, Germany), based on the Wilhelmy plate method. These surface tension measurements were performed at room temperature, and MilliQ water was used for instrument calibration. Calibration with water was repeated before each measurement. Three measurements of each sample were performed, and the average was calculated ± SD.

### 4.8. Dynamic Light Scattering (DLS) Determinations

The particle size analysis of HPβCD samples was performed by DLS using Nanotrac Wave (Microtrac Inc., Philadelphia, PA, USA). Several concentrations of aqueous HPβCD solutions were measured three times at 25 ± 0.5 °C. In order to study the possible influence of filtration and time on the formation/disassembly of aggregates, each CD sample concentration was divided in two samples. One sample was not filtered, but the other one was filtered through 0.45 µm RC membrane filter (Phenomenex, Cheshire, UK). All samples were immediately measured after preparation (t = 0 h), then stored at room temperature and measured again after seven days of storage. The intensity in percentage of each population was plotted versus the logarithm of size distribution.

### 4.9. Permeation Studies

Permeability studies of different HPβCD aqueous solutions (donor phase) were carried out in unjacketed Franz diffusion cells with diffusion area of 1.77 cm^2^ (SES GmbH—Analyse systeme, Bechenheim, Germany) operated at room temperature. Two ml of these aqueous HPβCD solutions were added to the donor compartment, while the receptor compartment was filled with 12 mL of MilliQ water. The receptor phase needed to be autoclaved before usage in order to remove all dissolved air and avoid the formation of air bubbles during the experiment. A single layer semi-permeable cellulose ester membrane (Biotech CE, Spectrum Europe, Breda, The Netherlands) with different molecular-weight-cutoffs (MWCO) was used to physically separate the compartments (i.e., the donor and receptor phases). The MWCO of membranes used were 3.5–5 kDa, 8–10 kDa, 20 kDa, 50 kDa, and 100 kDa. Depending on the aim of a given experiment, different sample times were used. For optimization of experimental conditions, 28 samples were collected from 5 to 360 min. For determination of aggregates in aqueous HPβCD solutions, samples were collected every 15 min for 75 min. A 150 µL sample (receptor phase) was collected at each time point and immediately replaced with same volume of receptor phase (i.e., MilliQ water). The receptor phase was agitated with a magnetic stirrer operated at 300 rpm. The samples collected were then quantified using the UHPLC validated method described previously. The calculation of steady state flux (J) of HPβCD was obtained from the slope (dq/dt) applying the linear regression relationship between time (t) and the amount of HPβCD in receptor chamber (q):

(7) $$J\;=\;\frac{\mathrm{dq}/\mathrm{dt}}{A}{\;=\;P}_{\mathrm{app}}\cdot C_{d}$$

in which dq/dt is the slope (in mg/s of CD permeated through the membrane over time, A is the diffusion area (1.77 cm^2^), and C_d_ is the total HPβCD concentration in the donor phase.

In order to assure sink conditions [40] were followed, both volume change and final concentration of components in donor phases were accessed.

The calculation of the apparent critical aggregation concentration for different tested membranes (i.e., concentration from which aggregates dimensions leads a deviation of ideal flux from CD through the membrane due to their size starts to be larger than the studied pore size) was performed by drawing tangent lines in the flux graphics. This deviation point where flux diversion from linearity started to be noticed corresponds to apparent cac value [21].

## Figures and Tables

**Table 1:** Intraday Precision and Percentage Recovery for HPβCD standard concentrations.

HPβCD Concentration (% *w*/*v*)	Intraday Precision		Accuracy
	(Average Peak Area ± SD)	%RSD	(Percentage Recovery ± SD)
0.5	17.41 ± 0.04	0.23	101.11 ± 0.45
2.5	18.17 ± 0.04	0.20	101.37 ± 0.20
5	17.41 ± 0.07	0.28	98.71 ± 0.27

**Table 2:** Calculated apparent cac values for HPβCDs in pure water and different semi-permeable membranes.

MWCO (kDa)	HPβCD
	cac% (*w*/*v*)
3.5–5	11.8
8–10	14.3
20	19.1
50	ND *
100	ND *

* not determined.

**Table 3:** Schematic table of main results, advantages, and disadvantages of assessed analytical methods.

Analytical Method	General Outcome	Advantages	Disadvantages
Osmometry	Positive deviation from linearity (solute–solvent interactions favored). Presence of HPβCD aggregate particles cannot be excluded.	Simple to perform. Sample dilution not required. Fast.	Low accuracy at low CD concentrations. Inadequate method for HPβCD aggregate quantification.
Viscometry	Exacerbated increase of viscosity deviating from linearity at CD conc. of 11% *w*/*v* (similar to sugars). Plausible method for detection/quantification of HPβCD aggregates in aqueous solutions.	Sample dilution not required. Fast.	This technique (plate method) can disassemble the aggregates by the mechanical forces involved.
Tensiometry	Addition of HPβCD to water has only minor effect on surface tension.	None.	Inadequate method for detection and quantification of HPβCD aggregates.
Dynamic Light Scattering	Uncertain results with available apparatus. Adequate method for detection of HPβCD aggregates.	Good approximation of aggregate size range.	Not suitable for aggregate quantification (i.e., to calculate the apparent cac value).
Permeation studies	Most useful and reliable method to detect and quantify (i.e., determine their apparent cac values) in aqueous HPβCD solutions.	Most accurate method with most precise results. Least “invasive” method for the aggregates (donor phase is always unstirred and quite still during experiment).	Time-consuming. Extremely laboring. Indirect results.
